# Kinetics, pathways, mechanism and toxicity evaluation of chiral pesticide Paichongding during the fermentation of Puer tea

**DOI:** 10.1016/j.fochx.2025.102300

**Published:** 2025-02-24

**Authors:** Shuai Qin, Tao Lin, Hen Tian, Lijian Du, Kaixi Li, Qiue Cao, Hongcheng Liu, Guofang Pang, Qiaoying Chang

**Affiliations:** a Institute of Quality Standards and Testing Technology, Yunnan Academy of Agricultural Sciences, Agricultural Product Quality Supervision and Inspection Center, Ministry of Agriculture, Kunming, Yunnan 650223, China; b School of Chemical Science and Technology, Yunnan University, Kunming 650000, China; c Chinese Academy of Inspection and Quarantine, Beijing 100176, China

**Keywords:** Paichongding, Puer tea fermentation, Metabolism, DFT

## Abstract

The presence of pesticide residues in tea has garnered increasing scholarly attention. Following the withdrawal of highly toxic pesticides from the market, there is a pressing need for alternative solutions to mitigate pest and disease occurrences in tea plantations. This study focuses on the neonicotinoid chiral pesticide of Paichongding(IPP), which possesses two chiral centers and four distinct isomers. We investigated the selective degradation of Paichongding isomers during the fermentation of Puer tea, tracking intermediate metabolites throughout the process. Using Density Functional Theory (DFT), we estimated the potential active sites of Paichongding and evaluated the toxicity of its intermediate products. Our findings indicate that Paichongding undergoes diastereo-selective degradation during Puer tea fermentation, resulting in the identification of seven key intermediates. Computational analysis revealed that the primary reaction sites of Paichongding are located on the imidazole and tetrahydropyridine rings. Toxicity assessments demonstrated that the metabolites exhibited heightened toxicity towards human genetic material, respiratory functions, and small aquatic organisms.

## Highlights

• This degradation pathway of Paichongding in Puer tea fermentation was firstly reported.
• Utilizing the density functional theory(DFT) for the structural identification of metabolic products.
• The toxicity assessment indicates that most metabolites have high toxicity to human respiratory system.

## Graphical abstract

**Figure:**
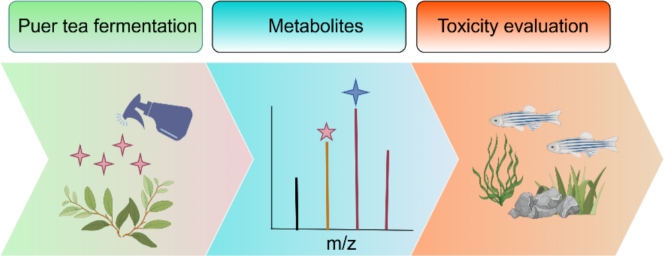

## 1 Introduction

Tea is one of the most popular non-alcoholic beverages in the world, among which Puer tea is a traditional Chinese famous tea, which belongs to post-fermentation tea, which is fermented by microorganisms (M. Lu et al., 2024). It faces challenges from pests and diseases in tea gardens, necessitating pesticide use. The shift from highly toxic pesticides to more degradable options has led to the prevalence of chiral pesticides, which make up about 40 % of the global pesticide market (Ji et al., 2023). Common chiral pesticides in tea cultivation include organophosphorus, organochlorine, pyrethroids, and neonicotinoids. Neonicotinoids mainly act on the nicotinoid acetylcholine receptors of insects (Casida, 2018), have been used over the past several decades to protect tea plants from pests such as leaf hoppers, aphids, whiteflies, and some lepidopteran species(Ge et al., 2021).

After tea leaves are harvested, the processing involves various transformations of chiral pesticides, which degrade at different rates depending on their isomers. Research by(Zhong et al., 2022) highlighted that R-indoxacarb residues decrease during the processing of green and black tea, while S-indoxacarb forms through chiral enantiomer transformation. Similarly,(Pan et al., 2015) noted the relative enrichment of (−)-acephate during the drying made of tea. As pesticides degrade during processing, they produce numerous intermediates, the toxicity and effects of which remain largely unknown. Notably, some metabolites, such as denitroylimidacloprid (a metabolite of imidacloprid), can be more toxic than their parent compounds (Klarich Wong et al., 2019). The neonicotinoid pesticide of Paichongding, which has two chiral carbons and four different isomers,has been studied for its degradation in soils with varying properties. Li, Huang, et al., 2016; Li et al., 2013; Li, Zhang, et al., 2016 found that selectivity exists between diastereomers, but not enantiomers, with trans isomers being more prone to mineralization. Further research by Cai, Zhang, et al., 2015 demonstrated that the biodegradation of Paichongding mainly occurs on the tetrahydropyridine ring, resulting in multiple metabolites. Additionally, Wang et al., 2013 observed that the SR and RS enantiomers of Paichongding were more readily absorbed by Chinese cabbage roots, leading to greater accumulation in edible leaves compared to RR and SS enantiomers. Fu et al., 2015; Fu, Wang, et al., 2013; Fu, Zhang, et al., 2013 showed that SR and RS degraded more efficiently in aerobic soils than their RR and SS counterparts, with major reactions including nitroso and propyl removal, redox reactions, and demethylation. Zhao et al., 2010 investigated the photolysis of Paichongding in water, identifying 28 photolysis products. Currently, there is no maximum residue limit (MRL) for Paichongding in tea, only the temporary MRLs in rice (0.5 mg/kg) or wheat (0.1 mg/kg). Paichongding with independent intellectual property rights is rapidly developed in China in recent years and was officially registered in 2017. It has been used in tea gardens. However, little information is available on enantiomers of Paichongding and its biodegradation intermediates on the fermentation of Puer tea. Puer tea is one of the most famous tea in Yunnan Province, China. Puer tea is processed as a post-fermented tea. The raw Puer tea is fermented by nature condition, its history is from long-distance transport with consign for horse. The ripen Puer tea is fermented by pile with high temperature (<50 °C) and high humidity condition. The flavor of ripened Puer tea was affected by lots of microbe. Although many studies about the chemistry and biological activities of Puer tea during fermentation are available in the literature, the effects of chiral pesticide degradation in Puer tea processing are still unclear and more complex (Liu et al., 2022).

In this study, the selective degradation of Paichongding during the fermentation of Puer tea was examined. This research tracked the trends of intermediate products and assessed their potential human and ecological risks, offering valuable theoretical guidance for managing this pesticide in terms of food safety across different contexts.

## 2 Materials and methods

### 2.1 Reagents and materials

Paichongding was sourced from Alta Technology Co., Ltd. (Tianjin, China), while ultra-pure water was prepared using the ELGACENTRA ultra-pure water system (Veolia, England). HPLC-grade acetonitrile and methanol were obtained from Merck (Darmstadt, Germany), and PSA and C_18_ materials were purchased from Dima Technology Co., Ltd. (Beijing, China). Various carbon nanotubes, including aminated and graphitized multi-walled types, were acquired from Xianfeng Nano Co., Ltd. (Nanjing, China). The sun-dried tea used in the study came from Xishuangbanna, Yunnan Province.

### 2.2 Experimental methods

To simulate the fermentation of Puer tea, 50 mg of the standard product was dissolved in 0.5 L of water and evenly sprayed onto the surface of 300 g of sun-dried green tea. The mixture was then tightly packed into a 500 mL flask and sealed with gauze to allow for air exchange. The flask was placed in an incubator for fermentation under controlled conditions. Sampling was conducted at 0, 1, 2, 3, 4, 5, 10, and 14 days. The moisture content was maintained at either 30 % or 40 %. A blank control, consisting of sun-dried green tea without the addition of pesticides, was also included for comparison.

### 2.3 Chromatographic separation

The four isomers of Paichongding were optimized using high-performance liquid chromatography (HPLC) with acetonitrile or methanol as the mobile phase, in combination with a chiral column. The study examined how variations in the mobile phase ratio and temperature affected the separation efficiency. In this context, k represents the capacity factor, where t_n+1_ and t_n_ are the retention times of two adjacent chromatographic peaks respectively, and α represents the separation factor. R represents the ideal gas constant, T is the absolute temperature, and ln∅ is the comparison. Δ*H* and Δ*S* denote the standard enthalpy and entropy changes of the analyte between the mobile and stationary phases. Additionally, ΔΔ*H* and ΔΔ*S* represent the differences in enthalpy and entropy changes between the enantiomers.

(1) $$k_{n}=\left(t_{n+1}-t_{n}\right)/t_{n}$$

(2) $$\alpha_{n}=k_{n+1}/k_{n}$$

(3) $$\ln k=-\frac{∆H}{\mathrm{RT}}+\frac{∆S}{R}+\ln\emptyset$$

(4) $$\ln\alpha =-\frac{∆∆H}{\mathrm{RT}}+\frac{∆∆S}{R}\;$$

### 2.4 Sample preparation

Weigh 2 g of tea samples into a 50 mL centrifuge tube, then add 10 mL of acetonitrile. Vortex the mixture for 1 min and allow it to stand for 2 h. Next, add 5 g of NaCl and vortex for an additional 2 min. Centrifuge the mixture at 5000 rpm for 5 min, and then transfer 2 mL of the supernatant into a new tube. Add 200 mg of PSA, 200 mg of C_18_, and 10 mg of multi-walled carbon nanotubes. After shaking and centrifuging at 5000 rpm for 1 min, collect 1 mL of the upper layer and filter it through a 0.22 μm filter for subsequent ultra performance liquid chromatography-high resolution mass spectrometry (UPLC-HRMS) or ultra performance liquid chromatography tandem mass spectrometry (UPLC-MS/MS) analysis.

### 2.5 Instrument analysis method

#### 2.5.1 UPLC-HRMS

The sample analysis was conducted using sample analysis was achieved in an ultra performance liquid chromatography-Q Exactive high resolution mass spectrometry (Thermo Fisher Scientific, Rockford, IL, U.S.A.) system. Chromatographic separation was achieved with a Hypersil GOLD column (100 × 2.1 mm, 1.9 μm) at 35 °C. The mobile phase comprised 95 % 0.1 % formic acid and 1 mmol ammonium in water, along with 5 % acetonitrile, at a flow rate of 0.3 mL/min.The gradient mobile phase was applied as follows: 0–2 min (5 % B), 2–27 min (5–95 % B), 27–32 min (95 % B), 32–33 min (95–5 % B), and 33–35 min (5 % B), with an injection volume of 1 μL. Positive calibration solutions were used, and the FS/DIA mode was employed, with FS scanning ranging from *m*/*z* 50 to 500 at a resolution of 35,000 FWHM. Parameters for AGC target and maximum IT were set at 3.0e6 and 100 ms, respectively.For DIA, the quality resolution was 17,500 FWHM, AGC target was 1e5, maximum IT was 50 ms, with a cycle count of 5 and an isolation window of 4 m/z. Step normalized collision energies (NCE) were set at 10 %, 20 %, and 30 %. The spray voltage was 3.5 kV for positive and 3.0 kV for negative modes. Sheath and auxiliary gas flow rates were 45 and 10 (units unspecified), respectively. Data analysis was performed using TraceFinder 4.1 EFS and Compound Discoverer 3.3 software.

#### 2.5.2 UPLC-MS/MS

The analysis utilized an Agilent 1290 ultra high-performance liquid chromatograph paired with an AB4500 series mass spectrometer from AB Sciex. Chromatography was performed on chiral amylopectin columns (3-chloro-5-methylphenylcarbamate) with dimensions of 250 × 4.6 mm and a particle size of 5 μm, maintained at 25 °C. The mobile phase consisted of 65 % 1 mmol ammonium acetate in water and 35 % acetonitrile, flowing at 0.8 mL/min, with a sample injection volume of 1 μL. The gradient mobile phase was as follows: 0–14 min (35 % B), 14–18 min (35–55 % B), 18–21 min (55–35 % B), and 22–25 min (35 % B). Multiple reaction monitoring (MRM) was conducted using positive ion electrospray ionization, targeting precursor ions at *m*/*z* 367.1, with product ions at 126.0* and 321.1. Concentrating/focusing potential was set at 92.7 V, while collision energies were 52.1 and 20.5 eV. The dry gas (N_2_) was heated to 550 °C with a flow rate of 8.0 L/min, optimizing ionization and detection efficiency.

### 2.6 Quality control

Three concentration levels (25, 62.5, and 125 μg/kg) of a single isomer of Paichongding, along with a matrix solution, were utilized to evaluate the detection limits and recovery rates. The chromatogram of the standardized sample, as well as the elution sequence of the racemates, was determined using an online optical rotation method (see Fig.S3). No residues of Paichongding were detected in the blank samples.The results demonstrated that the combination of multiple purification agents significantly enhanced purification efficiency, with multi-walled carbon nanotubes exhibiting particularly high recovery rates (refer to Fig.S4). The average recovery rates for the Paichongding racemates ranged from 78 % to 108 %. The relative standard deviation for accuracy was calculated to be between 4.3 % and 10.9 %.Within the linear range of 2.5 to 1250 μg/L, the correlation coefficient (R^2^) exceeded 0.99. The detection limit(LOD), defined as three times the signal-to-noise ratio (SNR), was determined to be 6.25 μg/kg, while the quantification limit(LOQ), defined as ten times the SNR, was established at 12.5 μg/kg (see TableS5 ∼ S6).

### 2.7 Degradation kinetics

In studies on the selective degradation of Puer tea, the degradation kinetics are typically modeled using a first-order kinetic framework:

(5) $$C=C_{0}e^{-\mathrm{kt}}\;\left(t_{1/2}=0.693/k\right)\;$$

(6) $$\mathrm{EF}=C_{\mathrm{RR}}/\left(C_{\mathrm{RR}}+C_{\mathrm{SS}}\right)\;$$

(7) $$\mathrm{DF}=\left(C_{\mathrm{RR}}+C_{\mathrm{SS}}\right)/\left(C_{\mathrm{RR}}+C_{\mathrm{SS}}+C_{\mathrm{SR}}+C_{\mathrm{RS}}\right)\;$$

Here, C_t_ represents the concentration of the compound at time, C_0_ is the initial concentration. EF is enantioselective and DF is diastereoselective.

### 2.8 Theoretical calculation

Based on the structural similarities between parent compounds and their respective degradation products, potential degradation pathways were inferred. Subsequently, density functional theory (DFT) calculations were performed using the B3LYP hybrid functional and the 6-311G(d,p) basis set, facilitated by the Gaussian09 software. The Fukui function was calculated using Multiwfn (version 3.8) (T. Lu & Chen, 2011), and isosurfaces were visualized with VMD (version 1.9.3). This theoretical framework allowed for a comprehensive elucidation of the degradation mechanism of Paichongding.

### 2.9 Toxicity assessment

The toxicity of intermediate products to human health was evaluated using ADMETLab (version 3.0) platform. By importing the structures (.mol files) into the Ecological Structure-Activity Relationship (ECOSAR) program (version 2.2),we estimated the acute and chronic toxicity of seven detected degradation products on aquatic organisms, including daphnia, fish, and green algae.

## 3 Results and discussion

### 3.1 Chromatographic separation

As shown in (Table S1 ∼ S2 and Fig.S2),under reversed phase conditions, acetonitrile exhibited superior separation performance compared to methanol. However, an increase in water content led to a gradual rise in the retention factor (k values). Additionally, as illustrated in (TableS3 ∼ S4 and Fig.S2) that with the increase of temperature, the values of retention factor k and separation factor α decreased, increasing temperature resulted in decreased values for both the retention factor k and the separation factor α. The separation degree (R_S_ values) initially increased before subsequently declining, with maximum values observed at 25 °C.Temperature variations impact the viscosity and dilution factor of the analyte; excessively low temperatures hinder mass transfer and diffusion of the solute, while excessively high temperatures facilitate these processes.

### 3.2 Degradation kinetics

**Fig. 1:**
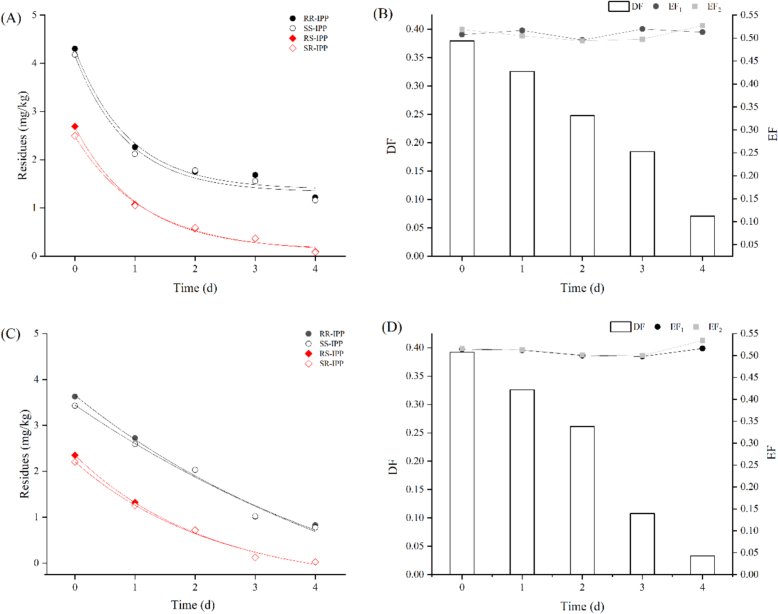
Degradation kinetics of Paichongding in Puer tea fermentation;(A) Mositure content of 30 % and (B) enantioselectivity of EF, DF; (C) Mositure content of 40 % and (D) enantioselectivity of EF, DF.

Following pesticide application, the post-fermentation process of Puer tea was conducted. The results indicated that Paichongding underwent rapid degradation during fermentation, consistent with first-order kinetics (Fig. 1). The half-lives of the various isomers ranged from 0.64 to 2.46 days, with shorter half-lives observed under high humidity conditions(TableS7).Moreover, diastereoselectivity was prominent during the degradation process, as the degradation rate of trans isomers exceeded that of cis isomers (Fig.S5), with nearly all trans isomers completely degraded by the fifth day. Therefore, it is recommended to utilize trans isomer monomers in tea gardens to minimize residue levels in the final product.

### 3.3 The stability of diastereoisomers

**Fig. 2:**
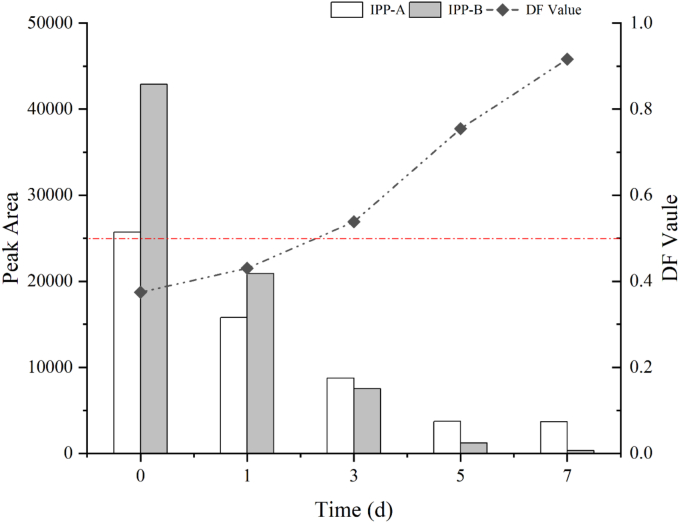
Degradation trend of diastereoisomers during fermentation of Puer tea.

Under a 70/30 acetonitrile/water solvent system, the diastereoisomers of Paichongding were successfully separated using a C18 column and subsequently incorporated into the Puer tea fermentation process. The results indicated that no interconversion occurred between the diastereoisomers, and that the trans isomers exhibited a degradation rate superior to that of the cis isomers (Fig. 2). These findings hold significant implications for the development of single-compound formulations and for minimizing pesticide residues in tea products.

### 3.4 Intermediates and possible degradation pathways

#### 3.4.1 Structural identification

By comparing the differences in metabolite profiles between pesticide-treated and blank fermentation samples over several days (Fig.S6), we employed a variety of analytical tools to extract metabolite information. Metabolite compounds were identified using the NIST17, HMDB20, GNPS21, GMD, and lipid MAPS databases, based on accurate mass measurements and retention times, which facilitated the determination of their molecular structures. For mass spectrometry imaging (MSI) annotations, we utilized mzCloud for online second mass spectrometry and ChemSpider for online first mass spectrometry, leveraging precise mass searches to verify and presumptively annotate metabolite signatures (Table 1).

**Table 1:** UPLC-HRMS data for Paichongding degradation products in Puer tea fermentation.

Product	Formula	RT (min)	Theoretical m/z [M + H]^+^	Measured m/z [M + H]^+^	Error(ppm)	Structure
IPP	C_17_H_23_ClN_4_O_3_	10.96,11.92	367.15314	367.15329	0.41	
TP325	C_14_H_17_ClN_4_O_3_	7.75	325.10619	325.10614	−0.14	
TP351	C_17_H_23_ClN_4_O_2_	11.05	351.15823	351.15833	0.28	
TP278	C_14_H_16_ClN_3_O	7.63	278.10547	278.10550	0.11	
TP339	C_15_H_19_ClN_4_O_3_	8.40	339.12184	339.12192	0.24	
TP264	C_13_H_14_ClN_4_O	6.90	264.09033	264.09021	−0.45	
TP276	C_14_H_18_ClN_3_O	12.03	276.08982	276.08997	0.54	
TP212	C_9_H_10_ClN_3_O	9.56	212.05852	212.05869	0.80	

The secondary mass spectrum of the Paichongding parent compound is illustrated in (Fig.S10). Notably, the fragment ion at *m*/*z* 321.1602 corresponds to the loss of a nitro group, while the ion at m/z 263.1184 results from the loss of a propoxy group. Among these, the ion at m/z 126.0100 consistently appears as a characteristic fragment ion.

For TP325, depicted in (Fig.S11), the retention time is 7.75 min, with a molecular formula of C_10_H_11_ClN_4_O and a [M + H]^+^ value of m/z 325.1061. The primary reaction involves the hydrolysis of the parent propoxy group to yield a hydroxyl group. Initially, *m*/*z* 308.1035 undergoes dehydration. Subsequently, the characteristic fragment ion m/z 279.1133 dissociates the nitro group from the tetrahydropyridine ring, while *m*/*z* 264.0898 loses a methyl group. The ion at *m*/*z* 126.0105 serves as a characteristic fragment of pyridine chloride.The concentration of TP325 is notably elevated during tea fermentation, exhibiting an initial increase followed by a decline, marking it as a principal metabolite in the fermentation process. Additionally, significant production of TP325 metabolites occurs in aqueous solutions across various pH levels, resulting in the near-complete hydrolysis of the parent Paichongding into TP325. The secondary fragment ions observed were consistent with those detected in tea, as shown in (Fig.S7 ∼ S8).

The peak time of TP339 was 8.40 min, detected at 8.40 min, this compound loses a nitro group and a methyl group, resulting in *m*/*z* 278.1059, indicative of alterations within the tetrahydropyridine ring (Fig.S12).

TP278 with a peak at 7.63 min, exhibits the loss of a methylene group leading to m/z 264.08997, alongside a characteristic fragment ion at *m*/*z* 126.00976, associated with pyridine chloride(Fig.S13).

At 11.05 min, TP351 undergoes nitro reduction to nitroso, accompanied by the loss of water (m/z 33.14786) and propoxy from the tetrahydropyridine structure (m/z 247.07462) (Fig.S14). Previous studies confirm its presence in aerobic soil (Cai, Wang, et al., 2015).

TP264 merges at 6.9 min, this newly identified metabolite involves the loss of one molecule of water (m/z 245.08084), suggesting it may represent a novel metabolic pathway in tea(Fig.S15).

Detected at 12.03 min, TP276 corresponds to m/z 258.07990, indicating the loss of water from a carbonyl-bound hydrogen in the tetrahydropyridine ring(Fig.S16), with previous reports of related metabolites in soil (Cai, Zhang, et al., 2015).

TP212,this metabolite, peaking at 9.56 min, reflects the loss of water from a carbonyl-bonded hydrogen on the imidazole ring (m/z 195.08879) (Fig.S17).

#### 3.4.2 Possible metabolic pathways of Paichongding in Puer tea

**Fig. 3:**
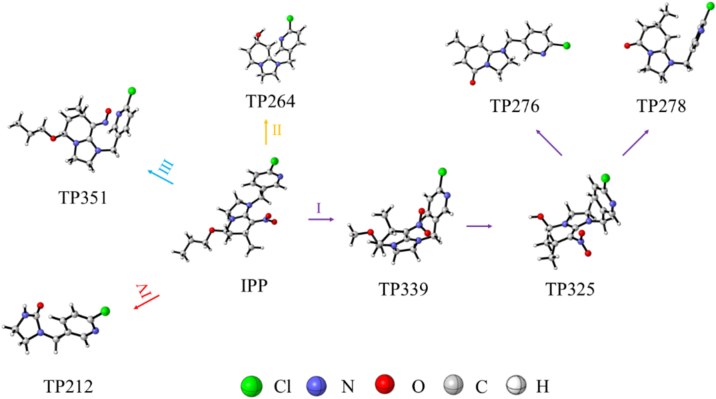
Proposed degradation pathway of Paichongding.

The potential metabolic pathways of Paichongding during the fermentation process of Puer tea were deduced (Fig. 3). One pathway involves the reduction of the nitroso group, while another entails the hydrolysis of the propoxy group to form a hydroxyl intermediate, which is subsequently oxidized to a ketone. A third pathway is characterized by the cyclomethyl hydroxylation of the tetrahydropyridine ring, and the final pathway involves the disruption of the entire tetrahydropyridine structure.

### 3.5 Degradation mechanism

**Fig. 4:**
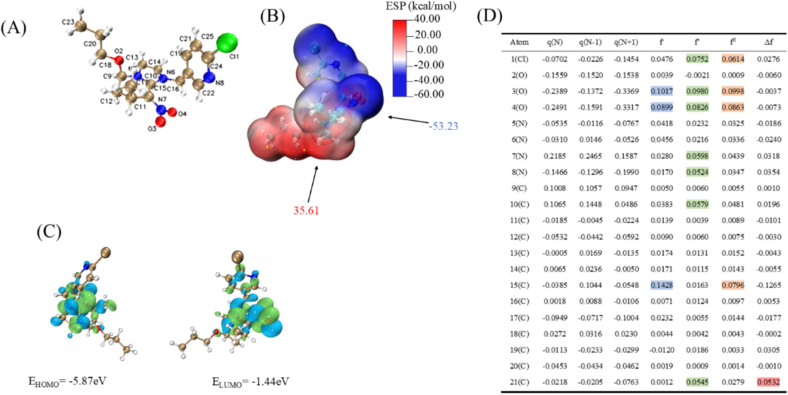
DFT calculations of Paichongding at the B3LYP/6–311G(d,p) level;(A) optimized structure, (B)ALIE surface, (C)HOMO-LUMO, (D) Fukui functions.

Through DFT calculation and quantitative analysis of molecular electrostatic potential(ESP), the minimum ESP value of Paichongding is −53.23 kcal/mol, which is located in the nitroso group region. ESP, with a maximum value of 35.61 kcal/mol, is located in the propoxygroup region, and the nitroso group region may be prefered to be attacked by electrophiles. In addition, highest occupied molecular orbital (HOMO) and lowest unoccupied molecular orbital (LUMO) energy levels differ by 4.43ev, a large energy difference indicating chemical instability. In addition, there are more HOMO and LUMO orbital components in imidazolium and tetrahydropyridine rings, so the reactions are concentrated in this region, while the chloropyridine region is difficult to react. By calculating the reduced Fukui function, it is shown that O(3) and O(4) are most vulnerable to electrophilic and free radical attacks (Fig. 4).

### 3.6 Toxicity evaluation

**Fig. 5:**
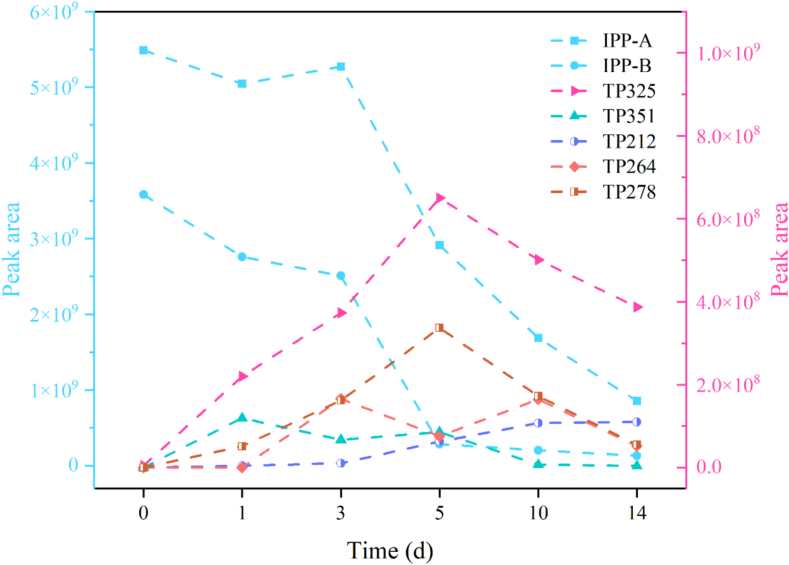
The variation trend of intermediate products in the fermentation process of Puer tea.

**Fig. 6:**
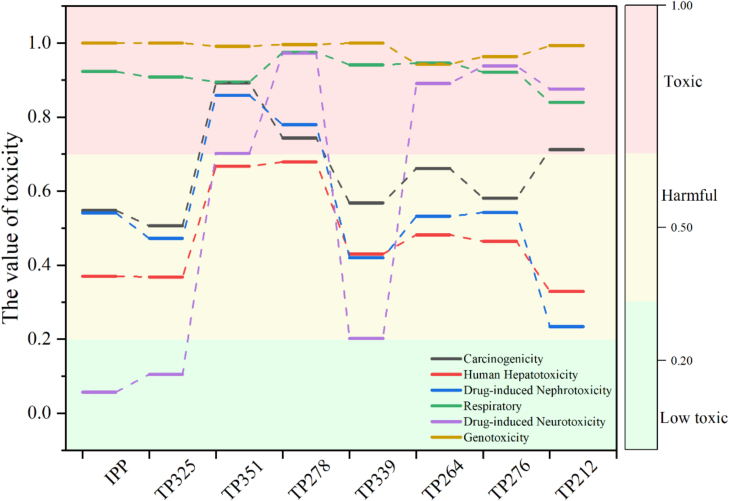
The predicted value of toxicity of intermediate products of Paichongding for human by ADMET Lab.

ADMET Lab platform was utilized to assess the toxicity of intermediates to humans, revealing that most exhibit significant genotoxicity to human respiratory systems, necessitating protective measures during field operations (Fig. 5). In the context of Puer tea fermentation, which typically lasts two months or longer, the trend of intermediate products indicates an initial increase followed by a decline (Fig. 6), suggesting a minimal health risk to humans. Furthermore, predictions made using ECOSAR software indicated that most intermediates are highly toxic to aquatic organisms, particularly daphnia (Fig.S9).

## 4 Conclusions

The predominant diastereoselective degradation of the chiral neonicotinoid pesticide Paichongding occurred during the fermentation of Puer tea, with the trans isomer exhibiting a faster degradation rate, thereby suggesting the development of the trans isomer monomer. Using UPLC-HRMS, seven intermediates were identified, with TP325 emerging as the principal metabolite. Theoretical calculations indicated that Paichongding predominantly reacts at the imidazolium and tetrahydropyridine rings. Computational toxicology assessments revealed that the intermediate poses significant risks to human respiratory, genetic, and neurotoxic health, as well as heightened toxicity to small aquatic organisms.

## CRediT authorship contribution statement

**Shuai Qin:** Writing – original draft, Software, Conceptualization. **Tao Lin:** Data curation. **Hen Tian:** Methodology, Investigation. **Lijian Du:** Investigation. **Kaixi Li:** Formal analysis. **Qiue Cao:** Writing – review & editing. **Hongcheng Liu:** Writing – review & editing. **Guofang Pang:** Writing – review & editing. **Qiaoying Chang:** Writing – review & editing.

## Declaration of competing interest

The authors declare that they have no known competing financial interests or personal relationships that could have appeared to influence the work reported in this paper.

## Data Availability

No data was used for the research described in the article.

## Appendix A Supplementary data

Supplementary material Image 9
