# Supplementary material for: Kinetics, pathways, mechanism and toxicity evaluation of chiral pesticide Paichongding during the fermentation of Puer tea
Source: Food Chem X. 2025 Feb 24;26:102300. doi: 10.1016/j.fochx.2025.102300 (PMC11919595; doi:10.1016/j.fochx.2025.102300)
Supplement: Supplementary file 1 — Supplementary material [file mmc1.docx]

**Table S1** Separation of Paichongding in different proportions of acetonitrile.

| ACN/H_2_O（v/v） | *k*_1_ | *k*_2_ | *k*_3_ | *k*_4_ | *α*_12_ | *α*_23_ | *α*_34_ | *R*_s12_ | *R*_s23_ | *R*_s34_ |
| --- | --- | --- | --- | --- | --- | --- | --- | --- | --- | --- |
| 40/60 | 1.23 | 1.33 | 2.82 | 3.12 | 1.08 | 2.12 | 1.11 | 0.42 | 6.18 | 1.13 |
| 35/65 | 1.78 | 1.92 | 4.29 | 4.73 | 1.08 | 2.23 | 1.10 | 0.64 | 9.26 | 1.48 |
| 30/70 | 2.81 | 3.02 | 7.20 | 7.91 | 1.07 | 2.38 | 1.10 | 0.85 | 12.65 | 1.69 |
| 25/75 | 4.84 | 5.14 | 13.53 | 14.63 | 1.06 | 2.63 | 1.08 | 0.81 | 15.55 | 1.58 |

| MeOH/H_2_O（v/v） | *k*_1_ | *k*_2_ | *k*_3_ | *k*_4_ | *α*_12_ | *α*_23_ | *α*_34_ | *R*_s12_ | *R*_s23_ | *R*_s34_ |
| --- | --- | --- | --- | --- | --- | --- | --- | --- | --- | --- |
| 90/10 | 1.22 | 1.38 | 2.06 | 2.16 | 1.31 | 1.49 | 1.05 | 0.95 | 2.49 | 0.28 |
| 85/15 | 1.58 | 1.80 | 2.83 | 2.98 | 1.14 | 1.57 | 1.05 | 1.14 | 3.33 | 0.35 |
| 80/20 | 2.13 | 2.44 | 4.06 | 4.27 | 1.15 | 1.66 | 1.05 | 1.24 | 4.04 | 0.39 |
| 75/25 | 2.98 | 3.43 | ̶ | ̶ | 1.15 | ̶ | ̶ | 1.26 | ̶ | ̶ |

**Table S2** Separation of Paichongding in different proportions of methanol.

**Table S3** The thermodynamic parameters of Paichongding.

| Linear equation | ∆*H*/  (kJ/mol) | ∆*S*/  (J/mol/K) | R^2^ |
| --- | --- | --- | --- |
| ln*α*_12_=49.02/T-0.0969 | -0.41 | -0.81 | 0.4361 |
| ln*α*_23_=549.02/T-0.9713 | -4.56 | -8.08 | 0.9980 |
| ln*α*_34_=31.89/T-0.0135 | -0.27 | -0.11 | 0.4833 |

**Table S4** The thermodynamic parameters of Paichongding.

| Linear equation | ∆∆*H*/  (kJ/mol) | ∆∆*S*/  (J/mol/K) | R^2^ | T_iso_/  (K) |
| --- | --- | --- | --- | --- |
| ln*k*_1_=427.07/T-0.4319 | -3.55 | -3.59 | 0.6860 | 988.86 |
| ln*k*_2_=467.13/T-0.4984 | -3.88 | -4.14 | 0.6842 | 937.20 |
| ln*k*_3_=1020.26/T-1.4841 | -8.48 | -12.34 | 0.8846 | 687.20 |
| ln*k*_4_=1049.94/T-1.4920 | -8.73 | -12.40 | 0.8757 | 704.03 |

| Compund | Spiked level  (μg/kg) | Recovery（%） | | | | | | | RSD(%) |
| --- | --- | --- | --- | --- | --- | --- | --- | --- | --- |
|  |  | 1 | 2 | 3 | 4 | 5 | 6 | Mean |  |
| RR-IPP | 25 | 88.4 | 86.3 | 97.8 | 101.4 | 82.5 | 99.8 | 92.7 | 8.6 |
|  | 62.5 | 87.6 | 88.4 | 79.8 | 85.3 | 92.2 | 86.0 | 86.6 | 4.7 |
|  | 125 | 71.5 | 73.5 | 79.5 | 82.1 | 80.1 | 84.8 | 78.6 | 6.5 |
| SS-IPP | 25 | 93.6 | 92.4 | 102.2 | 99.4 | 103.4 | 104.8 | 99.3 | 5.2 |
|  | 62.5 | 96.2 | 81.2 | 81.2 | 87.2 | 90.2 | 79.7 | 86.0 | 7.5 |
|  | 125 | 71.4 | 73.1 | 85.4 | 90.0 | 88.7 | 93.0 | 83.6 | 10.9 |
| RS-IPP | 25 | 92.5 | 89.4 | 99.4 | 97.8 | 99.4 | 93.7 | 95.4 | 4.3 |
|  | 62.5 | 94.8 | 89.0 | 81.5 | 100.0 | 78.0 | 79.8 | 87.2 | 10.2 |
|  | 125 | 88.8 | 93.1 | 106.6 | 115.2 | 114.0 | 106.0 | 104.0 | 10.4 |
| SR-IPP | 25 | 85.3 | 88.5 | 93.6 | 96.6 | 100.2 | 97.9 | 93.7 | 6.1 |
|  | 62.5 | 93.9 | 91.7 | 79.4 | 97.8 | 76.1 | 79.4 | 86.4 | 10.6 |
|  | 125 | 93.4 | 98.8 | 113.0 | 114.5 | 116.5 | 111.8 | 108.0 | 8.8 |

**Table S5** Recoveries and precision values for repeatability and reproducibility.

| Compund | Matrix | Linearity range(μg/L) | Regression equation | R^2^ | Matrix effect(%) | LOD  (μg/kg) | LOQ  (μg/kg) |
| --- | --- | --- | --- | --- | --- | --- | --- |
| RR-IPP | solvent | 2.5~1250 | *Y*=213*X*+9.12×10^3^ | 0.9994 | ̶ | ̶ | ̶ |
|  | tea | 2.5~1250 | *Y*=280*X*+1.80×10^3^ | 0.9937 | 31.45 | 6.25 | 12.5 |
| SS-IPP | solvent | 2.5~1250 | *Y*=222*X*+9.67×10^3^ | 0.9992 | ̶ | ̶ | ̶ |
|  | tea | 2.5~1250 | *Y*=269*X*+2.44×10^3^ | 0.9970 | 21.17 | 6.25 | 12.5 |
| RS-IPP | solvent | 2.5~1250 | *Y*=287*X*+9.45×10^3^ | 0.9999 | ̶ | ̶ | ̶ |
|  | tea | 2.5~1250 | *Y*=330*X*+2.69×10^3^ | 0.9984 | 14.98 | 6.25 | 12.5 |
| SR-IPP | solvent | 2.5~1250 | *Y*=314*X*+1.10×10^4^ | 0.9999 | ̶ | ̶ | ̶ |
|  | tea | 2.5~1250 | *Y*=322*X*+3.33×10^3^ | 0.9999 | 2.55 | 6.25 | 12.5 |

**Table S6** Method validation results for Paichonding in solvent and tea.

**Table S7** UPLC-MS/MS data for Paichongding degradation kinetics in Puer tea.

| Moisture content | IPP | Degradation rate  （%） | Residue  （mg/kg） | Equation  (C=C_0_e^-kt^) | R^2^ | DT_50_(days) |
| --- | --- | --- | --- | --- | --- | --- |
| 30% | RR-IPP | 71.67% | 1.22 | C=3.5767e^-0.282t^ | 0.8812 | 2.46 |
|  | SS-IPP | 72.27% | 1.16 | C=3.4692e^-0.287t^ | 0.8822 | 2.41 |
|  | RS-IPP | 96.46% | 0.10 | C=2.6628e^-0.776t^ | 0.9914 | 0.89 |
|  | SR-IPP | 96.58% | 0.09 | C=2.5957e^-0.780t^ | 0.9928 | 0.89 |
| 40% | RR-IPP | 77.20% | 0.83 | C=5.5486e^-0.754t^ | 0.8355 | 0.92 |
|  | SS-IPP | 77.38% | 0.78 | C=5.2327e^-0.732t^ | 0.8200 | 0.95 |
|  | RS-IPP | 98.75% | 0.03 | C=3.3847e^-1.069t^ | 0.9293 | 0.65 |
|  | SR-IPP | 98.84% | 0.03 | C=3.2734e^-1.077t^ | 0.9200 | 0.64 |


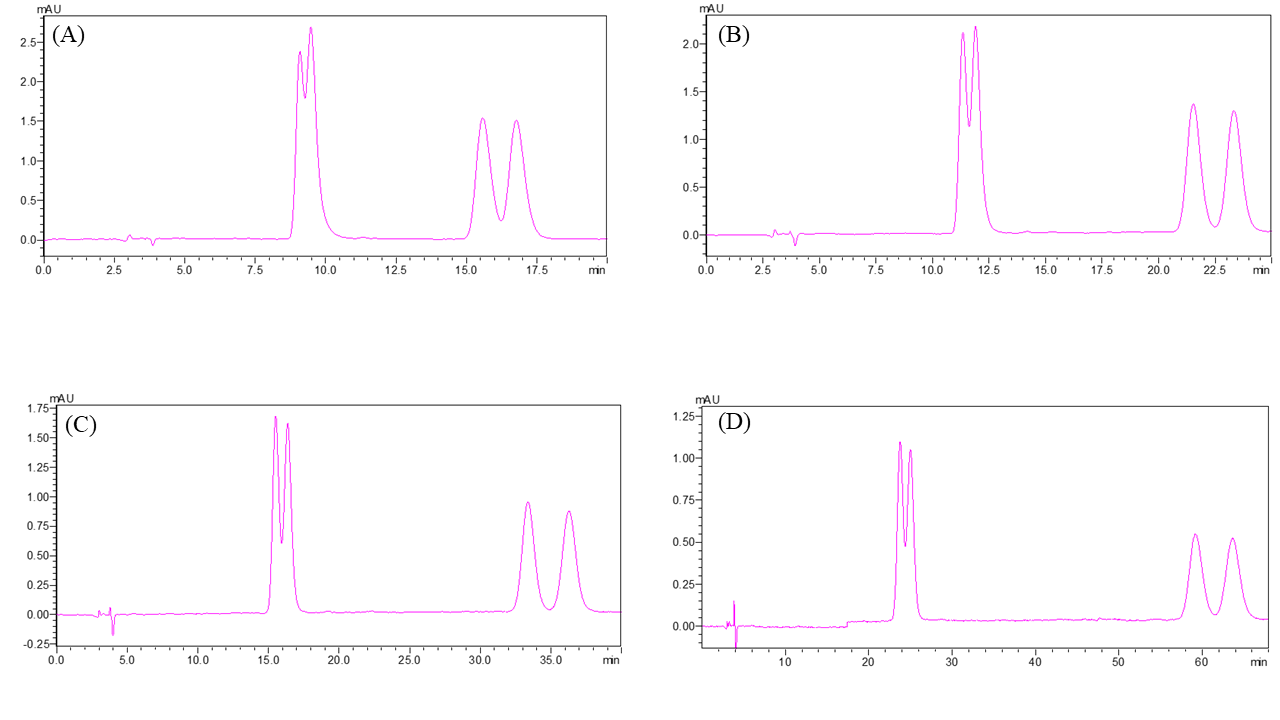


**Fig. S1**. The separation chromatogram of Paichongding at different ratios of acetonitrile.

**Fig. S2**. ln*α*-1/*T* and ln*k*-1/*T* chart of Paichongding.

**Fig. S3**. The chiral chromatogram and optical rotation of Paichongding.

**Fig. S4**. Effects of vortex times (A) and extraction solvents (B) and different kinds of sorbents (C) in Puer tea matrix.


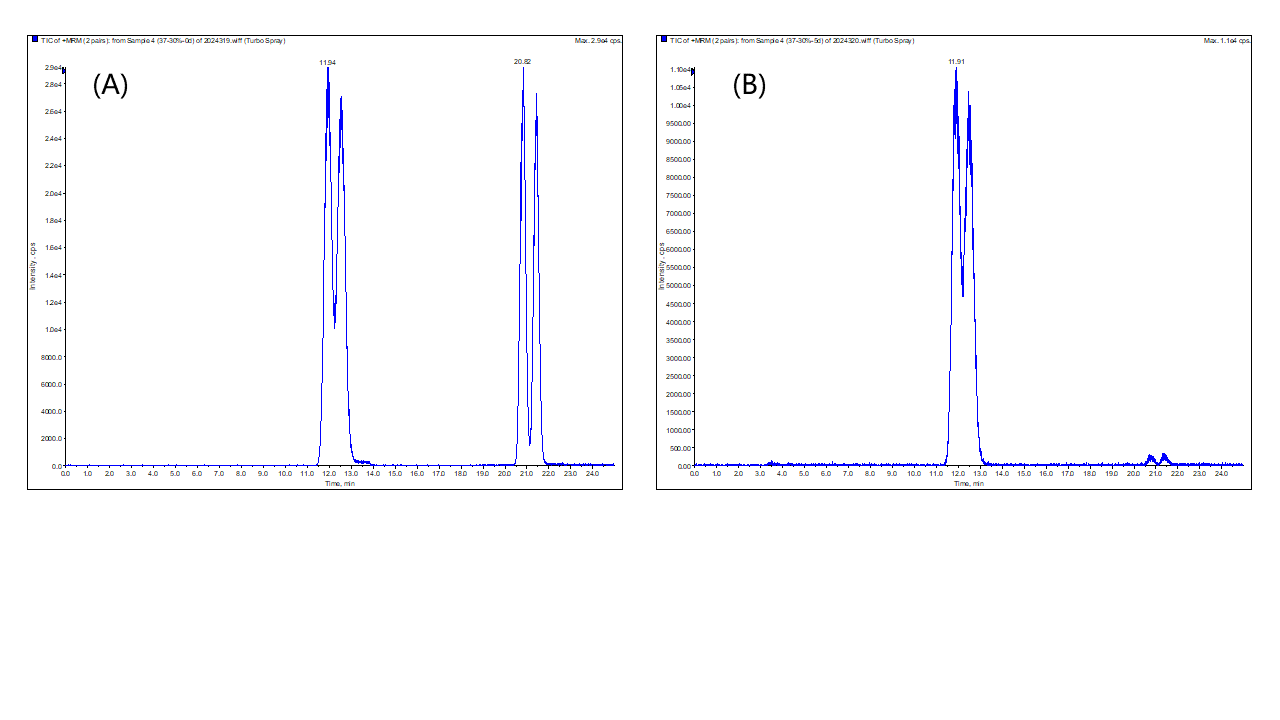


**Fig. S5**. Typical UPLC/MS chromatograms (A) 0d and (B) 5d of Paichongding degredation in Puer tea fermentation.

**Fig. S6**. UPLC-HRMS chromatograms (A) blank and (B) spieked pesticide of Paichongding degredation in Puer tea fermentation.


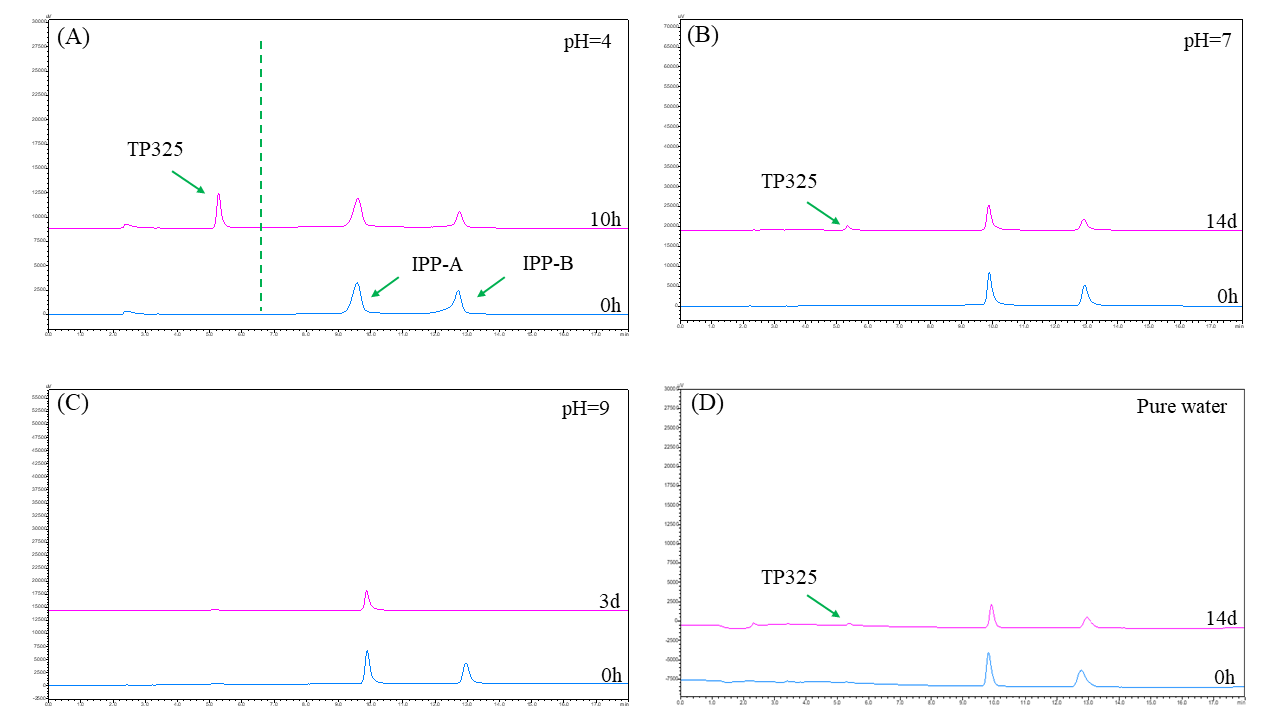


**Fig. S7**. The HPLC chromatograms of degradation of Paichongding in aqueous solutions at different pH.

**Fig. S8**. The mass spectrum of main metabolite (TP325) of Paichongding in water.

**Fig. S9**. The prediction of toxicity of Paichongding intermediates to non-target organisms in the environment by ECOSAR.

**Fig. S10**. The mass spectrum of parent (Paichongding) .

**Fig. S11**. The mass spectrum of TP325 .

**Fig. S12**. The mass spectrum of parent TP339 .

**Fig. S13**. The mass spectrum of TP278 .

**Fig. S14**. The mass spectrum of TP351 .

**Fig. S15**. The mass spectrum of TP264 .

**Fig. S16**. The mass spectrum of TP276 .

**Fig. S17**. The mass spectrum of TP212 .
